# Limited evidence that cancer susceptibility regions are preferential targets for somatic mutation

**DOI:** 10.1186/s13059-015-0755-5

**Published:** 2015-09-15

**Authors:** Mitchell J. Machiela, Brian M. Ho, Victoria A. Fisher, Xing Hua, Stephen J. Chanock

**Affiliations:** Division of Cancer Epidemiology and Genetics, National Cancer Institute, 9609 Medical Center Drive, Bethesda, MD 20892 USA

## Abstract

**Background:**

Genome wide-association studies have successfully identified several hundred independent loci harboring common cancer susceptibility alleles that are distinct from the more than 110 cancer predisposition genes. The latter are generally characterized by disruptive mutations in coding genes that have been established as ‘drivers’ of cancer in large somatic sequencing studies. We set out to determine whether, similarly, common cancer susceptibility loci map to genes that have altered frequencies of mutation.

**Results:**

In our analysis of the intervals defined by the cancer susceptibility markers, we observed that cancer susceptibility regions have gene mutation frequencies comparable to background mutation frequencies. Restricting analyses to genes that have been determined to be pleiotropic across cancer types, genes affected by expression quantitative trait loci, or functional genes indicates that most cancer susceptibility genes classified into these subgroups do not display mutation frequencies that deviate from those expected. We observed limited evidence that cancer susceptibility regions that harbor common alleles with small estimated effect sizes are preferential targets for altered somatic mutation frequencies.

**Conclusions:**

Our findings suggest a complex interplay between germline susceptibility and somatic mutation, underscoring the cumulative effect of common variants on redundant pathways as opposed to driver genes. Complex biological pathways and networks likely link these genetic features of carcinogenesis, particularly as they relate to distinct polygenic models for each cancer type.

## Background

The genetic basis of cancer susceptibility was first recognized in 1866 by the French neuroscientist Paul Broca, who noted clustering of breast cancer cases in his own family [1]. Generations of studies have observed an increase in the frequencies of cancer within families and between twins. In 1953, Nordling proposed that cancer is caused not by one but a number of mutations that are multiplied and accumulated over time [2]. Knudson further extended Nordling’s theory with his “two hit” hypothesis in which he proposed that retinoblastoma could develop due to an inherited germline mutation in combination with a somatic mutation [3]. While limited data were available when Nordling and Knudson first introduced their theories of the genetic basis of select cancers, recent cancer consortiums and technological advances have produced troves of data to explore the interplay between germline genetics and acquired somatic mutations.

Genome-wide association studies (GWAS) have generated a catalog of common susceptibility variants with small effect sizes that cumulatively contribute to sporadic cancer through a polygenic model [4, 5]. The combination of an agnostic analytical approach for scanning thousands of markers across the genome together with the scalability of studies drawn from different designs has accelerated the pace of discovery of markers, usually single nucleotide polymorphisms (SNPs) with minor allele frequencies greater than 5 %. Cancer GWAS have conclusively identified over 400 distinct susceptibility loci in over two dozen distinct cancers, including common cancers (e.g., breast, colon, and prostate) as well as rarer pediatric cancers (e.g., Ewing sarcoma and neuroblastoma) [6, 7]. To date, nearly all discovered susceptibility loci harbor many highly correlated SNPs, almost all mapping to the non-coding regions in genes, and roughly one-fifth have no nearby plausible candidate gene [7].

The Cancer Genome Atlas (TCGA) project along with other cancer genome sequencing initiatives, such as the International Cancer Genome Consortium, have emerged as indispensable resources for investigating the mutational landscape of cancer genomes [8, 9]. Utilizing next-generation sequencing technologies, these projects have mapped somatic mutations, localized copy number changes, and demonstrated that cancer genomes accumulate mutations over time; many of the mutations map to genes known to alter mechanisms that keep cellular proliferation in check [10].

An abundance of data exists on either cancer germline susceptibility alleles or somatic mutations, but little has been done to explore the interplay between germline genetics and somatic mutations in carcinogenesis. It is possible there is an overlap between germline cancer predisposing mutations and somatic cancer driver mutations. A prior investigation of cancer predisposition genes found that perhaps more than 40 % were oncogenic when mutated in tumor DNA [11]. The investigation also surveyed known cancer GWAS susceptibility loci at the time and found only 4 % of GWAS loci falling within cancer predisposition genes. For these cancer predisposition genes, none of the GWAS associated cancers matched the respective cancer subtypes that occurred in carriers of rare, high penetrant mutations, suggesting the mechanisms linking common, low penetrant alleles and rare, high penetrant alleles with cancer may be etiologically distinct.

Our goal was to investigate whether genes in cancer susceptibility regions harboring common variants with small estimated effect sizes have altered somatic mutation frequencies. Based on a literature search to aggregate published cancer susceptibility loci, we investigated somatic mutation frequencies using the cBioPortal database [12, 13] and TCGA in genes that fall within the intervals defined by the correlated variants discovered in cancer GWAS, and compared these mutation frequencies with expected cancer-specific background mutation frequencies. We explored further the relationship between germline susceptibility loci and somatic mutation frequency by examining mutation frequencies in a refined subset of genes shown to be pleiotropic across cancer types, affected by expression quantitative trait loci, or functionally important. Apart from a few notable exceptions, cancer-specific mutation frequencies for genes in susceptibility regions were not found to significantly differ from background mutation frequencies.

## Results

Results from the cancer GWAS literature search for each cancer subtype investigated are presented in Table 1. A total of 263 distinct germline susceptibility regions were reported as of 25 August 2014 and serve as the basis for this analysis. Breast and prostate cancer had the most discovered susceptibility regions with 80 and 71, respectively, after which were colon and skin cancer, each with 14 or more discovered susceptibility regions. Stomach and endometrial cancer had the fewest number of discovered susceptibility regions, each having fewer than five.

**Table 1:** Number of included cancer susceptibility regions and nearby genes for each cancer type investigated

Cancer subtype	GWAS loci	LD genes	Nearby genes
Bladder	12	103	264
Breast	80	702	1324
Cervical	7	148	290
Colon	25	215	421
Endometrial	1	1	18
Glioma	9	90	212
Kidney	5	12	48
Liver	7	89	195
Lung	10	257	350
Multiple myeloma	5	129	219
Ovarian	10	119	213
Prostate	71	697	1583
Skin	14	311	493
Stomach	2	12	24
Thyroid	5	15	40
Total	263	2190	4103

All cancer susceptibility regions have a published *p* value less than 5 × 10^−8^, are independent of each other, are associated with cancer in European populations, and were discovered prior to 25 August 2014. Linkage disequilibrium (LD) genes are those within the LD block of the susceptibility variant. Nearby genes are defined as those within the LD block of the susceptibility variant or within 500 kb of the LD block. For the genes, the total is for all unique genes and excludes duplicates across cancer types

Each cancer susceptibility variant was run through our analysis pipeline to generate a list of potentially affected genes that are within or near the linkage disequilibrium (LD) block defined by the combination of correlated SNPs and recombination hot spots that encompasses the susceptibility variant. The total number of genes is tabulated in Table 1. A total of 2190 unique genes are located in LD blocks of GWAS susceptibility loci and an additional 1913 are located ±500 kb of the LD blocks. A total of 24,482 genes are annotated in RefSeq Genes and 8.9 % of these genes thus fall within LD blocks of currently discovered variants for the cancer subtypes reported.

To investigate cancer subtype-specific background frequencies of mutation for genes falling within GWAS regions, we estimated cancer-specific frequencies of somatic mutation for all RefSeq genes. Across all cancers, the majority of RefSeq genes were not mutated; genes that did have mutations generally had frequencies lower than 4 % of individuals sampled. The top mutated gene for each cancer subtype is listed in Table 2. Examples of highly mutated genes include *APC*, *ERG*, *PTEN*, *TP53*, and *TTN*. Interestingly, none of these highly mutated genes map within or around LD blocks of cancer susceptibility regions for the respective cancer subtype.

**Table 2:** Top somatically mutated genes by cancer subtype

Cancer subtype	Samples	Most mutated (percentage samples)	GWAS locus
Bladder	328	*PIK3CA* (20.43)	No
Breast	1257	*TP53* (33.73)	No
Cervical	39	*TTN* (51.28)	No
Colon	224	*APC* (75.00)	No
Endometrial	248	*PTEN* (64.92)	No
Glioma	289	*IDH1* (76.12)	No
Kidney (chrom)	66	*MUC4* (66.67)	No
Kidney (clear)	522	*VHL* (46.55)	No
Kidney (pap)	168	*TTN* (25.00)	No
Liver	258	*TP53* (32.17)	No
Lung (adeno)	676	*TP53* (47.34)	No
Lung (small)	71	*TP53* (87.32)	No
Lung (squamous)	178	*TP53* (90.45)	No
Multiple Myeloma	205	*ADAM6* (30.73)	No
Ovarian (serous)	316	*TP53* (94.62)	No
Ovarian (small)	12	*SMARCA4* (91.67)	No
Prostate	584	*ERG* (29.87)	No
Skin	490	*TTN* (67.14)	No
Stomach	220	*TTN* (56.36)	No
Thyroid	401	*BRAF* (61.35)	No

**Fig. 1:**
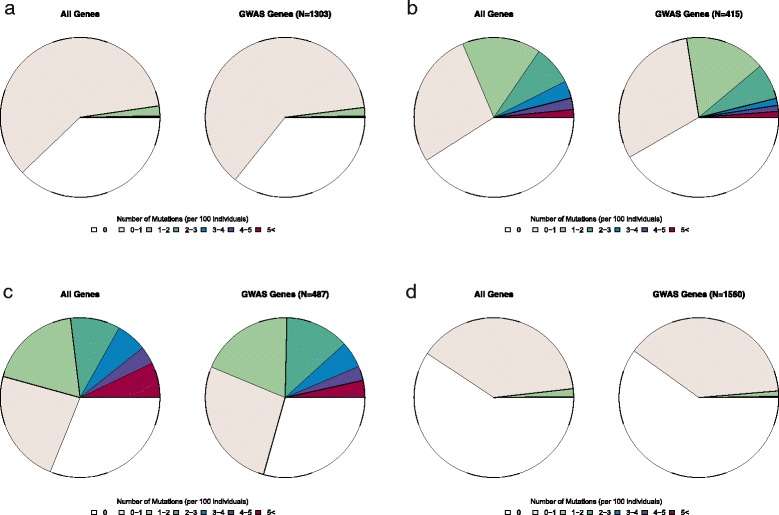
Mutations frequencies in cancer susceptibility regions compared with subtype-specific background mutation frequencies. Cancer subtypes displayed for breast (**a**), colon (**b**), melanoma (**c**), and prostate cancer (**d**)

Distributions of gene mutation frequencies within cancer susceptibility regions are compared with expected background frequencies of mutation for all RefSeq genes in Fig. 1. Differences in overall distribution of gene mutation frequencies were noted across cancer types and mirrored previously described cancer-specific mutation frequencies [14]. Skin, lung, colon, and cervical cancer subtypes were observed to exhibit higher background mutation frequencies than kidney, liver, or thyroid cancer. However, mutation frequencies for genes within or around LD blocks of cancer-specific susceptibility regions closely mirrored cancer subtype-specific background mutation frequencies. Only prostate and liver cancer displayed significantly different distributions for background and cancer susceptibility region mutation frequencies (Kolmogorov-Smirnov *p* < 0.05). Prostate cancer susceptibility regions were found to have a marginally lower mean gene mutation frequency than background (0.137 versus 0.154, *p* value = 0.038). Likewise, liver cancer susceptibility regions had lower mean gene mutation frequency than background (0.086 versus 0.321, *p* value < 0.001).

Highly mutated genes within LD regions of each cancer subtype are presented in Table 3. Overall, skin cancer had the highest somatic mutation frequencies for genes within cancer susceptibility regions, with *SORCS3*, *CDKN2A*, and *TRIOBP* all having mutations in over 10 % of samples. Bladder cancer, colon cancer, cervical cancer, and glioblastoma also had genes located in susceptibility regions with mutations in more than 10 % of samples. Cancers with low mutation frequencies in susceptibility regions include all subtypes of kidney, liver, multiple myeloma, and serous ovarian cancers; for each of these cancers, the somatic mutation prevalence was less than 5 %. Thyroid cancer had a particularly low somatic mutation frequency in susceptibility regions, with all genes having somatic mutations below 1 %.

**Table 3:** Top somatically mutated genes in cancer susceptibility regions

Cancer subtype	First	Second	Third	Fourth	Fifth
Bladder	*FGFR3* (14.939)	*EPG5* (3.049)	*TP63* (2.744)	*GIGYF2* (2.439)	*MECOM* (2.439)
Breast	*MAP3K1* (6.285)	*SYNE1* (4.773)	*BRCA2* (2.784)	*ZFHX4* (2.705)	*NOTCH2* (1.591)
Cervical	*COL11A2* (10.256)	*MED1* (10.256)	*EHMT2* (7.692)	*CDK12* (7.692)	*ABCF1* (5.128)
Colon	*HYDIN* (11.161)	*RYR3* (9.821)	*MAP3K4* (7.143)	*IGF2R* (6.696)	*MYO1B* (5.804)
Endometrial	*ACACA* (7.661)	*SYNRG* (2.823)	*TADA2A* (2.419)	*DUSP14* (1.613)	*C17orf78* (1.210)
Glioma (glioblastoma)	*EGFR* (20.235)	*PLEKHG4B* (1.760)	*CDKN2A* (1.466)	*HELZ2* (1.466)	*SLC6A19* (1.173)
Glioma (low grade)	*EGFR* (5.882)	*SLC6A3* (2.076)	*PLEKHG4B* (1.384)	*SLC6A19* (1.384)	*KMT2A* (1.384)
Kidney (chromophobe)	*ORAOV1* (1.515)	*PPFIA1* (1.515)	*SHANK2* (*1*.5*1*5)	NA (−.---)	NA (−.---)
Kidney (clear cell)	*ITPR2* (1.724)	*PPFIA1* (1.149)	*PRKCE* (0.766)	*ZEB2* (0.766)	*EPAS1* (0.575)
Kidney (papillary)	*ITPR2* (3.571)	*PPFIA1* (2.381)	*PRKCE* (1.190)	*ZEB2* (1.190)	*FGF3* (1.190)
Liver	*PIK3CD* (1.938)	*KIF1B* (1.938)	*CASZ1* (1.550)	*MYO1B* (1.550)	*CLDN8* (1.550)
Lung (adenoma)	*NOTCH4* (6.509)	*TNXB* (5.178)	*MDC1* (3.402)	*PLEKHG4B* (3.254)	*ZBED9* (3.254)
Lung (small cell)	*ZBED9* (7.042)	*SLC17A2* (5.634)	*BRD9* (4.225)	*SLC6A19* (4.225)	*HIST1H2AA* (4.225)
Lung (squamous)	*TNXB* (5.618)	*SLC6A3* (5.056)	*LRRC16A* (3.933)	*BTN2A2* (3.933)	*ZBED9* (3.933)
Multiple myeloma	*LTB* (1.951)	*NEU1* (1.951)	*DNAH11* (1.951)	*TNXB* (1.463)	*CACNA1I* (1.463)
Ovarian (serous)	*CPAMD8* (1.899)	*UNC13A* (1.266)	*MAST3* (1.266)	*HOXD10* (0.949)	*C10orf113* (0.949)
Ovarian (small)	*JAK3* (8.333)	NA (−.---)	NA (−.---)	NA (−.---)	NA (−.---)
Prostate	*SPOP* (7.877)	*SYNE1* (3.425)	*RYR1* (2.397)	*TNXB* (2.055)	*APOB* (1.712)
Skin	*SORCS3* (14.082)	*CDKN2A* (13.878)	*TRIOBP* (11.224)	*ANKRD11* (8.571)	*AOX1* (8.367)
Stomach	*ZBTB20* (7.273)	*MROH2B* (5.455)	*C6* (4.545)	*DRD3* (2.727)	*OXCT1* (2.727)
Thyroid	*TNS1* (0.748)	*TDRD7* (0.499)	*TBC1D2* (0.499)	*MBIP* (0.499)	*NKX2*-*1* (0.499)

Top gene name shown with percentage of samples mutated shown in parentheses

*NA* no additional mutated genes for cancer subtype

**Fig. 2:**
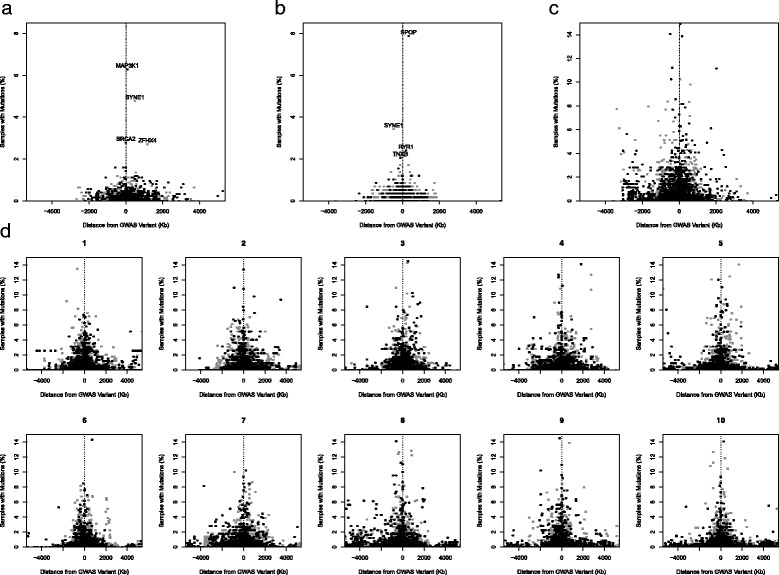
Frequency of gene mutations around cancer susceptibility variants. *Black points* are genes in LD blocks and *gray points* are genes outside LD blocks. Data for breast (**a**), prostate (**b**), all cancers (**c**), and random permutations (**d**) are plotted

**Fig. 3:**
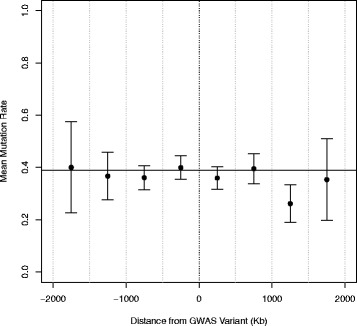
Mean gene mutation frequencies based on 500-kb bins. *Solid black line* is the average mutation frequency. *Points* are the mean bin mutation frequencies and *error bars* represent the 95 % confidence interval around the bin mean. *Dashed gray lines* are bin boundaries and the *dashed black line* is the location of the cancer susceptibility SNP relative to genes in the 500-kb bins

When we merged all genes from cancer susceptibility loci together and assessed their mutation frequency in relation to distance from the most associated susceptibility variant, we observed a pattern in which genes with higher mutation frequency tend to be closer in proximity to the most highly associated susceptibility variant (Fig. 2). This relationship was observed both within susceptibility regions of each cancer subtype (data shown for breast and prostate) and overall across cancer type. However, when comparing the distribution of gene mutation frequency for all cancer susceptibility regions to ten permutations of randomly selected array genotyped variants, the same overall pattern of a few genes in close proximity to the original susceptibility variant with higher mutation frequencies was observed. This suggests the observed relationship is not a function of genes near cancer susceptibility variants harboring higher mutation frequencies, but rather due to non-random placement of genes throughout the genome resulting in a higher density of genes in these susceptibility regions and thus a greater probability of outliers. To investigate if average frequencies of gene mutation are different based on distance from the susceptibility variant, 500-kb bins were constructed around susceptibility variants and mean frequencies of gene mutation were calculated (Fig. 3). No biologically relevant relationship was observed in gene mutation frequency in relation to distance from cancer susceptibility variant.

**Fig. 4:**
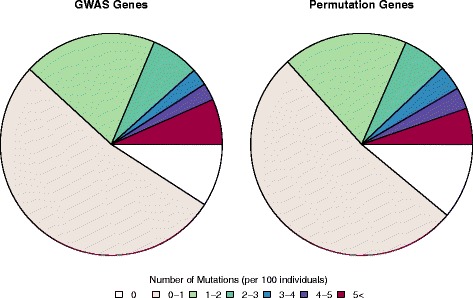
Top mutated gene for each cancer susceptibility region in cancers with more than 200 samples. *GWAS Genes* shows the observed distribution across all cancer types. *Permutation Genes* are a merged gene set across ten permutations of autosomal regions randomly selected throughout the genome

In an effort to remove the mutational signal of a possibly affected gene or genes at the cancer susceptibility loci from the background noise of other nearby but unaffected genes, the most mutated gene at each cancer susceptibility locus was selected and combined with all other top mutated genes in susceptibility regions. The analysis only included cancers with more than 200 sequenced cancer genomes, so stable estimates of mutation frequency were available. The distribution of gene mutation frequency for top mutated genes in cancer susceptibility regions was compared with distributions of top mutated genes from ten permutations of randomly selected SNPs. The observed distributions were remarkably similar with Kolmogorov-Smirnov tests indicating no significant differences with respect to GWAS susceptibility loci (Fig. 4).

**Fig. 5:**
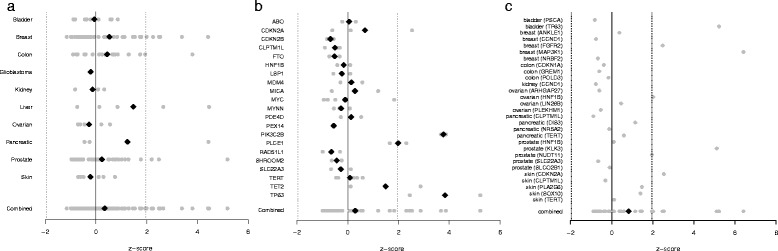
Normalized mutation frequency (z scores) of genes with varying levels of functional evidence. Analyses are shown for three overall gene classes: (**a**) pleiotropic genes, (**b**) eQTL genes, and (**c**) functional target genes. *Grey points* are mutation z score values and *black diamonds* are mean mutation frequencies

In an attempt to filter out potential functional genes in cancer susceptibility regions we performed a set of restricted analyses based on the level of functional evidence for each gene. Three classes of genes were of interest: (1) genes found to be in cancer susceptibility regions across multiple cancer subtypes, “pleiotropic genes”; (2) genes whose expression levels are associated with cancer susceptibility loci, “eQTL genes”; and (3) genes with experimental evidence linking a cancer susceptibility locus to a “functional gene”, based on laboratory investigation demonstrating an alteration in the regulation of one or more genes [15–30]. MutSigCV was used to find matching genes for each gene in these gene sets that have similar gene expression levels, DNA replication timing, and chromatin state, all of which are factors know to influence mutation frequency. For the pleiotropic gene analysis, results indicated no overall difference in mutation frequency z score (mean z score = 0.299, 95 % confidence interval (CI) = −0.074–0.672, *p* value = 0.11; Fig. 5a). Elevated frequencies of mutations were observed for *CDKN2A* (skin), *PIK3C2B* (breast, prostate), *PLCE1* (esophageal), *TET2* (breast), and *TP63* (bladder, lung). The eQTL gene analysis results also indicated no overall difference in mutation frequency z score (mean z score = 0.339, 95 % CI = −0.010–0.688, *p* value = 0.06; Fig. 5b). The highest mutation frequencies for eQTL genes were observed in *FGFR2*, *ITPR1*, *KIF13P*, *MAGI3*, *MGGT10*, *NOTCH2*, *SYNE1*, and *TACC2* for breast cancer, *MAP3K4* for colon cancer, *MYO1B* and *PIK3CD* for liver cancer, *IRX4* for pancreatic cancer, and *LMTK2*, *PDLIM5*, *SP4*, and *SYNE1* for prostate cancer. The analysis of functional genes found an overall increased mutation frequency (mean z score = 0.827, 95 % CI = 0.087–1.568, *p* value = 0.03; Fig. 5c). Of the 29 functional genes investigated, six had elevated mutation frequencies. These genes include *TP63* for bladder cancer, *FGFR2* and *MAP3K1* for breast cancer, *HNF1B* for ovarian cancer, *KLK3* for prostate cancer, and *CDKN2A* for skin cancer.

## Discussion

Our analysis of 263 published cancer susceptibility regions harboring common alleles, the majority of which were identified by GWAS, suggests that frequencies of somatically acquired mutations do not differ from background frequencies of gene mutation observed in the corresponding cancer. In other words, we did not observe evidence that common risk alleles appear to overlap with drivers of cancers. When refining our analysis to a subset of genes with functional evidence linking them to a susceptibility signal, our analysis indicates most of these target genes do not experience mutation frequencies that deviate from the expected. Except for a few notable examples, our observations suggest genes in regions harboring common germline susceptibility alleles do not exhibit an overall increase in mutation frequency, which is distinct from the more than 110 cancer predisposition genes [11].

The absence of an overall observed difference in mutation frequency at cancer susceptibility regions could be attributed to a signal-to-noise detection issue in which the methods we employed were not sufficiently sensitive to remove the signal of one to possibly two affected genes from a pool of potentially dozens of unaffected genes. To reduce the possibility that changes in mutation frequency were masked by statistical noise, we analyzed the data under several different analytical frameworks. First, frequencies of somatic mutation of all genes located in susceptibility regions do not deviate from expected background frequencies based on cancer subtype. Second, distributions of gene mutation frequency were similar when comparing susceptibility regions with ten permutations of random regions. Third, when restricted to the most highly mutated genes in intervals defined by susceptibility alleles in comparison to the randomly selected regions, no difference in distribution of somatic mutation frequency was detected.

The large genomic regions and number of genes covered by the spread of linkage disequilibrium with cancer susceptibility variants highlight the complexity of functionally mapping variants to their biological underpinnings. Regional and ancestry-specific differences in LD structure coupled with cell line-specific differences in chromatin patterns and receptor binding sites make it difficult to design high-throughput methods that are sensitive and specific enough to filter the large list of possibly affected genes. Focusing on genes implicated in multiple cancer subtypes as well as genes whose expression levels are influenced by cancer susceptibility loci are ways of enriching for genes that may be functional and thus may experience altered mutation frequencies [31, 32]. In addition, several focused efforts have been fruitful in identifying a handful of cancer susceptibility regions where the affected gene has been determined [15–30]. When we compared sets of polygenic genes, eQTL genes, and functionally mapped genes with covariate matched genes, a modest overall enrichment for elevated mutation frequency was observed; however, this enrichment was only statistically significant for the functionally mapped genes (*p* = 0.03). In each set, a subset of genes experienced elevated mutation frequencies, whereas the majority of genes in these regions demonstrate no elevated mutation frequencies. These observations suggest that if the functional genes of cancer susceptibility alleles do have elevated mutation frequencies, then the increases are minimal for most and do not overlap with established drivers of cancer.

The observation of no notable overall change in somatic mutation frequency in genes within cancer susceptibility regions could perhaps be explained as follows. Most cancer-associated variants are relatively common, have low estimated effects, and are often located in regulatory elements that cause minor changes in gene expression (e.g., the bladder cancer rs2978974 locus and *PSCA* expression levels [16]). It is also plausible that genes targeted by cancer susceptibility regions might affect other host factors — for example, the cells responsible for the immune response to tumors — and we may, therefore, be looking at mutation frequencies in the wrong tissue type. Purifying selection is expected to remove deleterious variation from the gene pool, but is less effective at removing cancer susceptibility variants with minor effects, allowing such variants to reach common frequencies in human populations, particularly since most cancers occur well after the age of reproduction. On the contrary, somatic mutations across cancer genomes often cluster in important genes regulating cellular growth, cell cycle checkpoints, and DNA repair. These mutations act as driver mutations (e.g., oncogenes or tumor suppressor genes) with highly deleterious effects, usually leading to a downstream cascade of later mutations in other important genes. Differences in mutational frequency in cancer susceptibility target genes and cancer predisposition genes might reflect the level of functional importance of these genes in maintenance of normal cellular integrity. For example, cancer predisposition genes that are often somatically mutated may be located at highly conserved cores of essential biological pathways, whereas cancer susceptibility target genes with average mutation frequencies might be genes with a high degree of functional redundancy. As a result, a somatic mutation of a cancer susceptibility functional gene (defined by a common, low-effect variant) may be neither sufficient nor necessary to lead to cancer development, as evidenced by the low relative risks observed in GWAS. Still, the accumulation of many small effects of common alleles appears to account for a substantial fraction of the genetic risk for sporadic adult cancers; the steady cataloging of common susceptibility alleles supports the contribution of a polygenic risk model, involving many small perturbations of redundant pathways, as suggested by our data.

## Conclusions

There is likely a complex interplay between germline genetics and somatic mutations. The germline can alter cancer risk over time due to small perturbations in many key, redundant pathways, some of which could permit escape of dangerous somatically altered cells. Somatic mutations occurring in important oncogenes or tumor suppressor genes may serve as a necessary hit required to drive the process of carcinogenesis. Apart from a few exceptions, our analysis suggests genes nearby common germline susceptibility variants do not display overall increased somatic mutation frequencies, unlike the cancer predisposition genes. Future work focused on understanding the biological basis of cancer susceptibility alleles will be instrumental in better understanding the complex interplay between germline genetics and somatic mutations.

## Materials and methods

A literature search of PubMed was performed to identify all reports of cancer susceptibility studies published before 25 August 2014. These publications were merged with reports from the National Human Genome Research Institute’s Catalog of Published Genome-wide Association Studies [6] to arrive at a comprehensive list of genetic variants associated with germline susceptibility to cancer. Further filtering was performed to remove variants with association *p* values greater than 5 × 10^−8^, highly correlated variants in high LD, and variants discovered in populations of non-European ancestry.

An analysis pipeline using custom Python scripts (Python 2.7.5 [33]) was developed to extract potential genes of interest around cancer susceptibility regions (available at [34] through the MIT license). First, LD blocks were defined based on European recombination frequency data from HapMap Phase 2 [35]. These frequencies were estimated from phased haplotypes in HapMap release 22 (NCBI 36) for the CEU population and are publically available for download [36]. We defined LD blocks as all genomic positions neighboring the tagging susceptibility variant that are within recombination frequency peaks of 20 cM/Mb or higher. Second, a window of interest around the tagging susceptibility variant was extended by 500 kb in both directions beyond the LD block boundaries to ensure the inclusion of additional genes potentially regulated by the susceptibility region since cancer susceptibility variants may have functional effects on genes that are outside the LD block. Third, genes were extracted that overlap the window of interest around each significant cancer susceptibility locus. We utilized the RefSeq Gene [37] database publically available on the UCSC FTP site [38]. For genes with multiple transcripts, inclusion in the susceptibility window of interest was based on the start coordinate of the transcript with the earliest start position and the end coordinate of the transcript with the latest stop position.

The analytical pipeline generated lists of putative genes altered or regulated by one or more variants residing in the LD block of a GWAS associated tagging SNP. Each gene was investigated for mutations using available tumor genomes from databases. The frequency of mutations per gene of several cancer types was extracted from the cBioPortal database [12, 13, 39]. Tumor genomes were available from Asan Medical Center (AMC) [40], Beijing Genomics Institute (BGI) [41, 42], British Columbia Cancer Research Centre [43], BROAD Institute [44–49], Cornell University [47, 48], Clinical Lung Cancer Genome Project (CLCGP) [50], Genentech [51], Johns Hopkins University [52], Memorial Sloan-Kettering Cancer Center (MSKCC) [53–55], International Cancer Genome Consortium (ICGC), RIKEN [56], Sanger Institute [57], TCGA [58–68], Tumor Sequencing Project (TSP) [69], University of Michigan [70], and Yale University [71]. We queried tumor sequencing data through the CGDS-R package using R version 3.0.1 “Good Sport” [72].

To estimate cancer-specific background frequencies of mutation, mutation frequencies for all RefSeq genes were queried for each cancer subtype. Background mutation frequencies were compared with frequencies of mutation for genes within cancer susceptibility regions to investigate differences in mutation frequency. Statistical significance was assessed by two-sample Kolmogorov-Smirnov tests. Furthermore, to estimate the expected distribution of gene mutational burden across cancer genomes, we performed random sampling throughout the genome. For each cancer type, a random autosomal SNP present on the commercially available Illumina 660 W-Quad genotyping platform was chosen to represent each significant cancer susceptibility allele marked by one or more SNP variants. Randomly chosen SNPs were analyzed using the same pipeline, the genes in the LD region were extracted, and mutational frequencies for the genes were queried in cBioPortal. To provide statistical robustness, ten permutations of this procedure were performed for each cancer type. Distributions from each of the ten permutations were compared with that of the observed mutational distribution of the cancer type to assess for significant differences in mutational frequency.

Several cancer susceptibility regions have at least some level of evidence linking a gene to a cancer susceptibility signal. The first set of genes with a higher probability of being functional is a set of “pleiotropic genes” which we define as any gene that was associated at genome-wide significance levels (*p* < 5 × 10^−8^) with more than one cancer subtype. A second set of interest is “eQTL genes”, which are genes whose expression levels are affected by a cancer susceptibility variant. These eQTL genes are filtered out by performing eQTL analyses for all genes in the LD window plus 500 kb around cancer susceptibility variants. Publically available TCGA expression and genotyping data were used in combination with linear regression models to determine if there was significant evidence for an eQTL. If the cancer susceptibility variant of interest was not directly genotyped, a genotyped variant in high LD (R^2^ > 0.6) was used as a surrogate. Finally, a set of “functional genes” was extracted from a literature search. Functional genes were defined as any gene with at least one publication linking a cancer susceptibility locus to a gene with experimental evidence [15–30]. To explore whether these sets of functionally enriched genes had altered frequencies of somatic mutation, lists of genes with similar expected background mutation frequencies were generated using MutSigCV [14]. Genes were matched based on transcriptional activity, DNA replication timing, and chromatin state. Lists of up to 50 matching genes were generated for each functional gene. Cancer-specific frequencies of mutation were extracted from cBioPortal, and mutation z scores were calculated based on means and standard deviations of matching gene sets.

All plotting and statistical analyses were performed in R version 3.0.1 “Good Sport” [72] on a 64-bit Windows platform. Statistical tests and reported *p* values are two-sided.

### Data availability

All datasets used to assess tumor mutation frequency are publically available at cBioPortal. Cancer subtype-specific accession codes are as follows: bladder (blca_mskcc_solit_2012, blca_bgi, blca_tcga_pub, blca_tcga); breast (brca_bccrc, brca_broad, brca_sanger, brca_tcga_pub, brca_tcga); cervical (cesc_tcga); colon (coadread_genetech, coadread_tcga_pub, coadread_tcga); endometrial (ucec_tcga, ucec_tcga_pub); esophageal (esca_broad); glioma (glioblastoma: gbm_tcga_pub2013, gbm_tcga_pub, gbm_tcga; glioma: lgg_tcga); kidney (chromophobe: kich_tcga; clear: kirc_bgi, kirc_tcga_pub, kirc_tcga; papillary: kirp_tcga); liver (lihc_amc_prv, lihc_riken); lung (adeno: luad_broad, luad_tcga_pub, luad_tcga, luad_tsp; small: sclc_clcgp, sclc_jhu; squamous: lusc_tcga_pub, lusc_tcga); multiple myeloma (mm_broad); ovarian (serous: ov_tcga_pub, ov_tcga; small: scco_mskcc); pancreatic (paad_icgc, paad_tcga); prostate (prad_broad_2013, prad_broad, prad_mskcc, prad_tcga, prad_mich); skin (skcm_broad, skcm_tcga, skcm_yale); stomach (stad_tcga); and thyroid (thca_tcga).

## Abbreviations

CI: confidence interval
eQTL: expression quantitative trait locus
GWAS: genome-wide association study
LD: linkage disequilibrium
SNP: single nucleotide polymorphism
TCGA: The Cancer Genome Atlas
